# Physical and geometric determinants of transport in fetoplacental microvascular networks

**DOI:** 10.1126/sciadv.aav6326

**Published:** 2019-04-17

**Authors:** Alexander Erlich, Philip Pearce, Romina Plitman Mayo, Oliver E. Jensen, Igor L. Chernyavsky

**Affiliations:** 1 School of Mathematics, University of Manchester, Oxford Road, Manchester M13 9PL, UK.; 2 Department of Mathematics, Massachusetts Institute of Technology, 77 Massachusetts Avenue, Cambridge, MA 02139-4307, USA.; 3 Centre for Trophoblast Research, Department of Physiology, Development and Neuroscience, University of Cambridge, Cambridge CB2 3EG, UK.; 4 Homerton College, University of Cambridge, Cambridge CB2 8PH, UK.; 5 Maternal and Fetal Health Research Centre, Division of Developmental Biology and Medicine, School of Medical Sciences, University of Manchester, Manchester Academic Health Science Centre, Manchester M13 9PL, UK.

## Abstract

Across mammalian species, solute exchange takes place in complex microvascular networks. In the human placenta, the primary exchange units are terminal villi that contain disordered networks of fetal capillaries and are surrounded externally by maternal blood. We show how the irregular internal structure of a terminal villus determines its exchange capacity for diverse solutes. Distilling geometric features into three parameters, obtained from image analysis and computational fluid dynamics, we capture archetypal features of the structure-function relationship of terminal villi using a simple algebraic approximation, revealing transitions between flow- and diffusion-limited transport at vessel and network levels. Our theory accommodates countercurrent effects, incorporates nonlinear blood rheology, and offers an efficient method for testing network robustness. Our results show how physical estimates of solute transport, based on carefully defined geometrical statistics, provide a viable method for linking placental structure and function and offer a framework for assessing transport in other microvascular systems.

## Teaser

Key geometric factors that determine nutrient transport in the functional exchange unit of the human placenta are identified.

## INTRODUCTION

The human placenta performs diverse functions later taken on by several different organs (*1*). In particular, it mediates the exchange of vital solutes, including respiratory gases and nutrients, between the mother and the developing fetus. The complex heterogeneous structure of the placenta is adapted to perform these various functions. However, despite its availability for ex vivo perfusion experiments just after birth and the importance of placental dysfunction in conditions such as fetal growth restriction, the link between placental structure and function in health and disease remains poorly understood (*2*, *3*). Multiscale models have proved successful in investigating aspects of the structure-function relationship in the microcirculation (*4*, *5*), lymph nodes (*6*), and organs including the brain (*7*–*10*), the kidney (*11*), and the liver (*11*, *12*). However, general methods for incorporating experimental data on complex, heterogeneous capillary networks into these models remain underdeveloped.

**Fig. 1:**
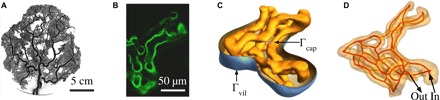
Multiscale structure of the fetoplacental vasculature. (**A**) Fetoplacental arterial vessels [imaged using micro x-ray tomography; reproduced with permission via CC-BY from (*16*)] deliver blood from the umbilicus through numerous bifurcating vessels to peripheral capillary networks [e.g., (**B**), imaged using confocal microscopy]. The fetoplacental vasculature is confined within villous trees that are coated with syncytiotrophoblast and are bathed in maternal blood; capillary networks sit within terminal villi, the peripheral branches of the trees. (**C**) A segmented confocal image of a terminal villus reveals the surface Γ_cap_ of fetal capillaries (yellow) and the surrounding syncytiotrophoblast (blue; Γ_vil_) that interfaces with maternal blood. Image processing yields capillary centerlines (red) (**D**), which have total length *L*_c_. The assumed inlet and outlet vessels are indicated. Fetal blood occupies the volume Ω_b_ confined by Γ_cap_; villous tissue occupies the space between Γ_cap_ and Γ_vil_.

Recent advances in three-dimensional (3D) imaging have revealed aspects of placental structure in intricate detail (Fig. 1) (*13*–*16*). Fetal blood flows from the umbilical cord through a complex network of vessels that are confined within multiple villous trees; the trees sit in chambers that are perfused with maternal blood. Much of the solute exchange between maternal and fetal blood takes place across the thin-walled peripheral branches of the trees (terminal villi), which contain the smallest fetoplacental capillaries. Quantitative measurements have demonstrated structural differences between healthy and pathological placentas (such as changes in villous capillary network density) (*17*), but physical explanations for the observed symptoms of diseases such as preeclampsia and diabetes have so far been confined mainly to analyses of diffusive conductances from 2D histological data (*17*–*21*). Here, we establish how the elaborate and irregular 3D organization of capillaries within the terminal villi, the primary functional exchange units of the fetoplacental circulation, contributes to solute exchange.

To maximize functional understanding from emerging 3D structural data requires an integrated mix of ex vivo experiments (*22*, *23*) and computational modeling (*14*, *20*, *24*–*28*). Previous studies have demonstrated how transport of highly diffusive solutes in capillaries with small diffusion distances is flow limited (determined by the flow rate of fetal or maternal blood), whereas transport of slowly diffusing solutes in capillaries with a thick villous membrane is diffusion limited. While research has begun to shed light on the relationship between these transport regimes in the human placenta (*14*, *26*, *29*), the latest imaging data allow for a much more comprehensive characterization of the dominant geometric features and physical processes that govern solute transport in the placental microvasculature. Quantifying these structure-function relationships is essential in building well-grounded multiscale models for whole-organ function of the human placenta and other complex vascular systems (*6*, *10*, *12*, *24*, *30*).

In this study, we use an integrative approach. We combine image analysis and 3D simulations with a discrete network model and asymptotic analysis to examine the dependence of solute transport on the geometrical arrangement of capillaries within the terminal villi. The properties of these functional exchange units are quantified and encapsulated in a theory of fetoplacental transport (formulated as an algebraic relationship) that links the complex 3D structure of fetal microvascular networks to their solute exchange capacity, providing a valuable building block for organ-level models. We test the reduced scaling relationship against image-based computations and find that it applies both at the level of the whole network and within individual capillaries (subject to variations due to countercurrent effects), readily incorporating non-Newtonian effects of whole blood. Our results suggest that an archetypal physical scaling of fetoplacental solute transport based on geometrical statistics provides a viable method for linking placental structure and function. Furthermore, our developed and cross-validated framework offers significant savings in computational costs associated with image-based models of complex biological structures and could be applicable to other systems in which transport occurs via advection and diffusion in disordered microscale networks.

## RESULTS

### Theory of solute transport in fetoplacental networks

The terminal villus shown in Fig. 1C is one of four samples we analyzed, obtained by confocal laser scanning microscopy [from (*14*, *25*)]. Even within a single villus, there is significant variation in capillary diameters and exchange distances between the capillary and villous surfaces (see fig. S1). Image segmentation (section S1) reveals the domains occupied by blood vessels (Ω_b_) and villous tissue (Ω_t_), as well as the bounding syncytiotrophoblast, which provides an interface Γ_vil_ with maternal blood. For each sample, identifying likely inlet and outlet vessels, we computed Stokes flow through the vessel network in Ω_b_ (non-Newtonian features of blood rheology are addressed below) under an imposed pressure drop Δ*P* to determine the network resistance R (Table 1). Solute transport was computed using a linear advection-diffusion equation in Ω_b_ (modifying the advection term by a factor *B* to account for facilitation of solute transport by red blood cells), coupled to a diffusion equation in Ω_t_: Solute concentrations differing by a value Δ*c* were prescribed on Γ_vil_ and the inlet to Ω_b_, and the net flux *N* of solute out of Ω_b_ was evaluated. Solute uptake by tissue is not accounted for in this study. Full details of the simulations are provided in section S2.

**Table 1:** Geometric parameters for network specimens 1 to 4. The viscous resistance scaled by the blood viscosity and the diffusive length scale specific to the villus (integrated ratio of exchange area over exchange distance) are determined computationally (see section S2); the total centerline length is determined through a skeletonization algorithm of the capillary network that provides vessel centerlines.

**Specimen**	**1**	**2**	**3**	**4**
R/η × 10^7^[mm^−3^]	7.4	3.5	27.9	28.0
L [mm]	8.2	11.4	15.4	17.9
*L*_c_ [mm]	2.2	1.8	2.2	2.3
L/*L*c	3.7	6.5	7.0	7.7

**Fig. 2:**
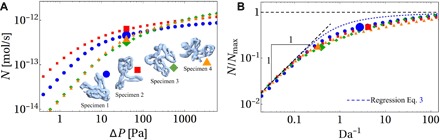
Predictions of solute flux versus transport parameters. Computational data (A) show appreciable collapse when plotted using suitable dimensionless variables (B). (**A**) Computed solute flux *N* in four segmented villus networks (specimens 1 to 4) plotted against the pressure drop Δ*P* driving flow through each network. (**B**) The same data presented in terms of the inverse Damköhler number (see Eq. 2) and solute flux scaled on each specimen’s diffusion-limited upper bound *N*_max_. Da^−1^ is proportional to the pressure drop driving flow through the network. Predicted fluxes for each specimen (small colored symbols) collapse toward a common relationship. Dashed lines show the approximation Eq. 3 and its asymptotes. For specimen 1, the largest deviation between the approximation Eq. 3 and the computational result is 24%. The large symbols in (A) and (B) compare fluxes in each specimen evaluated at a fixed inlet-outlet pressure drop Δ*P* = 40 Pa. We consider this value of Δ*P* physiological as it leads to shear stresses in specimen 1 below approximately 1.2 Pa, which we identify in section S2 to be a physiological value.

For each of the four specimens (illustrated in fig. S1A), the computed net solute flux (evaluated using parameter values appropriate for oxygen) rises monotonically with the imposed pressure drop (Fig. 2A). We wish to establish how the differing structures of each network lead to differences in the relationship between *N* and Δ*P*. This understanding is facilitated by identifying the relevant dimensionless parameters and variables describing transport in this functional tissue unit (*30*).

Flow-limited transport arises when Δ*P* is sufficiently weak for solute to be fully saturated in fetal blood before it leaves the vessel network. In this case, *N* is determined by the flow rate through the outlet (Δ*P*/R) as *N* = Δ*cB*Δ*P*/R (where *B* models facilitated transport). In contrast, an upper bound on *N* arises when the transport is diffusion limited, with flow being sufficiently rapid to impose the fixed concentration difference Δ*c* between Γ_vil_ and the boundary Γ_cap_ (the capillary endothelium separating Ω_b_ from Ω_t_). In this case, *N* = *N*_max_ ≡ *D*_t_Δ*c*L, where L is a length scale specific to the villus and *D*_t_ is the solute diffusivity in tissue (*3*). (L can be evaluated by solving Laplace’s equation ∇^2^*c* = 0 in Ω_t_, with *c* = 0 on Γ_cap_ and *c* = Δ*c* on Γ_vil_, and integrating the normal gradient of *c* over either Γ_cap_ or Γ_vil_; see section S2.) We can compare the diffusive capacity per unit concentration across the villous tissue, *D*_*t*_ L, with a dimensionally equivalent measure of diffusive capacity along vessels using the dimensionless parameter

$$\mu =\frac{D_{t}L}{D_{p}L_{c}}$$ (1)

where *D*_p_ is the solute diffusivity in blood plasma and *L*_c_ is a measure of vessel length in the villus. Taking *L*_c_ as the total centerline length of capillaries within the network, it is notable that the ratio L/*L*_c_ shows only modest variation between specimens (Table 1), despite significant variability in network structure (Fig. 2A, insets).

The ratio of fluxes in the diffusion- and flow-limited states defines a dimensionless Damköhler number

$$\text{Da}\;=\frac{D_{t}LR}{B\Delta P}$$ (2)

which also has an interpretation as a ratio of a time scale for advection within the vessel network to a diffusive time scale through the tissue. The parameters μ and Da are convenient for characterizing solute exchange in a terminal villus (*3*), as illustrated for a single vessel in section S3.

For each villus sample, we computed three geometric determinants of transport: *L*_c_, L, and R/η (in simulations, we used uniform blood viscosity η = 2 × 10^−3^ Pa∙s; see Table 1). The R and L values are larger for specimens 3 and 4 than for specimens 1 and 2, likely because the latter were fixed at approximately three times higher fetal perfusion pressure (see Materials and Methods). It is notable that differences revealed by these global measures are not obviously captured by simpler summary statistics such as average capillary radii (fig. S1).We then replotted the relation between net flux *N* and pressure drop Δ*P* in terms of *N*/*N*_max_ (scaling flux on the diffusion-limited upper bound) and Da^−1^ (the natural dimensionless proxy for Δ*P*). These variables incorporate dependencies on the material parameters *B*, *D*_t_, and *D*_p_, which we report for different solutes in table S1. Despite substantial variation in network structure, the data collapse appreciably (Fig. 2B), showing a common smooth transition between flow- and diffusion-limited transport as Da^−1^ increases. The large symbols in Fig. 2 show how, at a fixed physiological inlet-outlet pressure drop Δ*P* = 40 Pa (section S2), geometric differences in flow resistance between specimens lead to different inverse Damköhler numbers Da^−1^ (Fig. 2B).

Extending a regression formula proposed previously (*3*, *26*), we approximate the relationship between *N* and Da^−1^ (section S3) using

$$N=\frac{N_{\text{max}}}{\text{Da}{\left(1-e^{-\text{Da}}\right)}^{-1}+{\text{Da}}_{F}^{1/3}}$$ (3)

which captures the simulated fluxes with a reasonable degree of accuracy (Fig. 2B). Here, the parameter ${\text{Da}}_{F}=\mu^{2}\text{Da}/\alpha_{c}^{3}$, where α_c_ ≈ 5.5, accounts for transport across concentration boundary layers within capillaries (*26*). Setting this term aside for a moment, the remaining terms provide a smooth transition between flow-limited transport (*N* ≈ *N*_max_/Da when Da^−1^ ≪ 1) and diffusion-limited transport (*N* ≈ *N*_max_ when Da^−1^ ≫ 1; Fig. 2B). Despite substantial variation in network structure, the data collapse toward a common relationship (Fig. 2B) in the flow-limited (Da^−1^ ≪ 1) and diffusion-limited (Da^−1^ ≫ 1) regimes while showing similar qualitative behavior in the transitional region for Da = *O*(1).

**Fig. 3:**
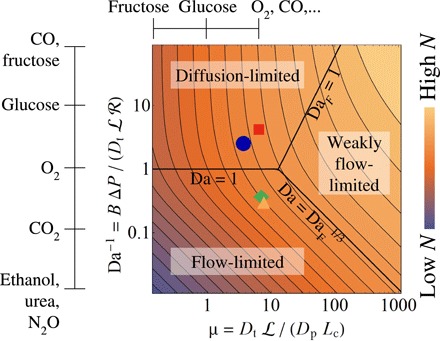
A diagram summarizing transport regimes in the parameter space spanned by μ (see Eq. 1), measuring the tissue’s capacity for diffusive transport relative to diffusion in the vessel network, and Da^−1^ (see Eq. 2), which is proportional to flow. Contours and background color indicate the network solute flux *N* (see Eq. 3), evaluated for fixed Δ*c* and *L*_c_. The diffusion-limited regime [Da^−1^ ≫ max(1,μ^2^)], for which *N* ≈ *N*_max_, and two flow-limited regimes are indicated. In the strongly flow-limited state [Da^−1^ ≪ min(1,μ^−1^)], flux is proportional to flow (*N* ≈ *N*_max_Da^−1^), corresponding to an asymptote shown in Fig. 2B. In the weakly flow-limited state (μ^−1^ ≪ Da^−1^ ≪ μ), concentration boundary layers arise within capillaries and *N* ≈ *N*_max_Da^−1/3^/μ^2/3^. The large colored symbols correspond to those in Fig. 2, placing oxygen transport in specimens 1 to 4 outside the weakly flow-limited regime, spanning the interface of strongly flow- and diffusion-limited regimes. Vertical and horizontal bars outside the figure indicate the relative μ and Da^−1^ values of a variety of solutes with respect to oxygen based on data in table S1. The upper limits of the ranges of table S1 are shown. For instance, Da^−1^ of glucose is approximately 10 times higher compared with oxygen, and μ of glucose is approximately 10 times lower.

This transition is illustrated on the left-hand side of the regime diagram in Fig. 3. The symbols show how, imposing a physiological inlet-outlet pressure drop Δ*P* = 40 Pa across all four specimens, oxygen fluxes span the transition between flow- and diffusion-limited states. Equation 3 suggests that, for villi and solutes having sufficiently large μ (i.e., rapid transmural diffusive transport), boundary layer effects may emerge (*26*), introducing an intermediate weakly flow-limited state for intermediate values of Da. However, our simulations demonstrate that, for oxygen transport in the four samples investigated, μ is sufficiently small for this not to be relevant under normal conditions. Figure 3 also shows that, between different specimens, Da spreads over more than an order of magnitude, for a given Δ*P*, reflecting differing flow resistances among villi. In contrast, the ratio L/*L*_c_ and, hence, the parameter μ (Eq. 1) vary by approximately a factor of 2, as revealed by Table 1.

We can extend this analysis to a variety of small and mobile solutes using the data in table S1, which summarizes estimated effective advection enhancement factors *B*, plasma diffusivities *D*_p_, and tissue diffusivities *D*_t_. From these, we compute inverse Damköhler numbers relative to the value for oxygen. Taking oxygen transport as a reference, we identify strongly diffusion-limited solutes, such as mannitol, fructose, or carbon monoxide (for which ${\text{Da}}_{\text{rel}}^{-1}\gg 1$), as well as strongly flow-limited solutes, including certain anesthetic gases (e.g., nitrous oxide), urea, and ethanol (for which ${\text{Da}}_{\text{rel}}^{-1}\ll 1$). It is noteworthy that the transport regime in which a solute lies (see Fig. 3) is affected by the inverse Damköhler number through the ratio *B*/*D*_t_ and affected by the diffusive capacity ratio μ through the ratio *D*_t_/*D*_p_. As table S1 shows, for a fixed geometry, Da has a much wider spread than μ through large variability of *B*, which ranges over four orders of magnitude. However, the maximum achievable flux *N*_max_ is proportional to *D*_t_ alone, and therefore, *N*_max_ values for oxygen and CO are predicted to be almost twice those of ethanol and caffeine for the same transmural concentration difference (table S1).

### Network heterogeneity

**Fig. 4:**
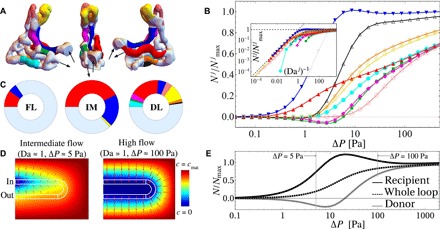
Solute exchange heterogeneity at the level of individual capillaries. (**A**) The nine longest capillaries of specimen 1 are highlighted in color; the rest of the network is shown in light blue. Arrows indicate inlet and outlet in three projections of the network. The blue capillary near the inlet neighbors the green and magenta capillaries near the outlet; likewise, red (near inlet) neighbors orange and black (near outlet). (**B**) Scaled net uptake of vessel *j*, $N^{j}/N_{\text{max}}^{j}$, as a function of the pressure drop Δ*P* across the whole network exhibits nonmonotonicity in some cases, due to a donor-recipient mechanism explored in (D) and (E). The inset shows a log-log plot of the same data as a function of (Da^*j*^)^−1^, highlighting a collapse of the data similarly to the whole network (Fig. 2B), with the exception of donor capillaries for which *N* becomes negative (truncated curves). (**C**) Relative contributions of different capillaries to the net uptake of the entire network. The inlet-outlet pressure drop in the flow-limited (FL) regime is Δ*P* = 0.04 Pa, in the intermediate (IM) regime is Δ*P* = 1.26 Pa, and in the diffusion-limited (DL) regime is Δ*P* = 186 Pa. (**D**) Simplified capillary loop model system of donor-recipient mechanism, from a computation in two spatial dimensions. Red arrows illustrate directions of diffusive flux in the surrounding tissue; capillary boundaries are shown as white lines. At intermediate pressure drops, a countercurrent effect extracts solute from the bottom capillary (acting as a donor) into the top capillary (recipient). The net fluxes of the inlet recipient and outlet donor capillaries as a function of pressure drop (**E**) show the same characteristic behavior as demonstrated in (B): At intermediate pressure drops, the donor(s) switch sign, whereas the recipient surpasses its carrying capacity *N*_max_, but this effect is integrated out at the level of the whole system (whole loop).

To understand spatial variations in solute transfer within capillary networks, we now focus on solute exchange at the level of individual capillaries. For the nine longest capillaries of specimen 1 (highlighted in Fig. 4A and labeled by *j*), we evaluated the scaled net uptake, $N^{j}/N_{\text{max}}^{j}$, as a function of the pressure drop Δ*P* across the whole network (see the log-linear plot in Fig. 4B). The scaled net uptake exhibits heterogeneity across the sample of vessels, including nonmonotonicity in some cases. In particular, uptake in the blue capillary surpasses its carrying capacity *N*_max_ at intermediate Δ*P*. Conversely, transport in the neighboring magenta and green capillaries switches sign around the same intermediate pressure-drop regime, suggesting a change in their role from donors of oxygen at low Δ*P* to recipients at high Δ*P* (via a mechanism explored in Fig. 2, D and E). The inset shows a log-log plot of the same data as a function of (Da^*j*^)^−1^, highlighting a collapse of the data similarly to the whole network (Fig. 2B), with the exception of donor capillaries for which *N* becomes negative (truncated curves).

To illustrate the donor-recipient mechanism, we consider a simplified model system in Fig. 4D. A capillary loop, embedded in a box of villous tissue, carries solute from the inlet (top) to the outlet (bottom) capillary. At intermediate pressure drops, a countercurrent effect extracts solute from the outlet capillary (acting as a donor) into the inlet capillary (the recipient). The net flux of the top and bottom capillaries as a function of pressure drop (Fig. 4E) shows the same characteristic behavior as demonstrated in Fig. 4B: At intermediate Δ*P*, the donor flux switches sign, whereas the recipient surpasses its carrying capacity *N*_max_. At the level of the entire loop, however, the net uptake *N* neither surpasses the carrying capacity *N*_max_ nor becomes negative. Similarly, the heterogeneity seen in individual vessels of the specimen 1 capillary network (Fig. 4B) is integrated out at the level of the entire network (Fig. 2A).

Clarification of the donor-recipient mechanism adds to our understanding of the contributions of individual vessels to the overall solute transfer of the capillary network, as shown in Fig. 4C. For a low inlet-outlet pressure drop, the network is situated in the flow-limited regime, where practically all uptake is reduced to a narrow region near the inlet. Among the nine colored capillaries, only the blue and red ones are close to the inlet, adding a small contribution each. In the intermediate regime, the donor-recipient effect peaks, favoring the blue recipient capillary at the expense of the neighboring green and magenta donors from which solute is extracted (and, to a lesser extent, the red at the expense of orange and black). In the diffusion-limited regime, capillaries at the periphery of the network, in proximity to a large portion of the surrounding villous surface (particularly the red and yellow capillaries), add the greatest contributions to transport. Figure 4C therefore illustrates how different vessels contribute to transport as the network moves from a flow-limited to a diffusion-limited state across Fig. 3.

**Fig. 5:**
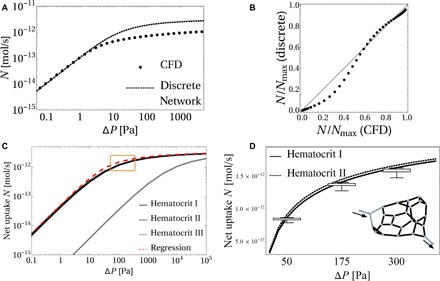
A comparison between the discrete network versus CFD models of oxygen transfer in specimen 1. [topology shown as an inset to (D)]. (**A**) Comparison of the solute flux *N* versus network pressure drop Δ*P* as predicted by the computational model (section S2) and the discrete network model (section S4). (**B**) Same data when rescaled by relevant values of *N*_max_. (**C**) Dependence of the discrete network-predicted oxygen net transfer rate *N* on hematocrit distribution. The oxygen transfer rate for varying Δ*P* for the entire network is predicted assuming uniform hematocrit and facilitated transport (*B* = 141, hematocrit I, solid line), spatially variable hematocrit [*B* = *B*(*H*), hematocrit II, dashed line], and uniform hematocrit but without facilitated transport (*B* = 1, hematocrit III, thin dashed line). The regression equation Eq. 3 applied to the entire discrete network is shown as the red dashed line (see section S4). (**D**) Sensitivity of net oxygen flux to removal of individual vessels. The solid curves replicate those within the orange box in (C). For three different pressure drops (Δ*P* = 50, 175, and 300 Pa), we calculated 33 values of *N* with each of the 33 black capillaries (inset) removed individually. The resulting distribution for the nonuniform hematocrit model is shown with box plots, demonstrating that the network is robust with respect to the occlusion of individual capillaries.

The computational results underlying Figs. 2 to 4 are based on a Newtonian transport model with uniform hematocrit, evaluated using 3D finite-element simulations. To assess the non-Newtonian effects of hematocrit on solute transport, we developed a discrete network model (see section S4) that relies on the well-established semiempirical Pries-Secomb model (*31*), implemented in a reduced representation of each network in which each capillary is treated as a discrete component (section S4). Figure 5 (A and B) compares predictions of the reduced (discrete network) model to the full [computational fluid dynamics (CFD)] model for uniform hematocrit and blood viscosity. Although the discrete network model captures the scaling relationship between the uptake flux *N* and the pressure drop Δ*P* (Fig. 5C) and shows a good overall agreement with the CFD (Fig. 5, A and B), the discrete network model overestimates *N* at large Δ*P* and underestimates *N* at small Δ*P* (see Discussion for further context). Figure 5C compares the net oxygen transfer, assuming either uniform hematocrit and blood viscosity (hematocrit I, where *H* = 0.48, η = 2 × 10^−3^ Pa∙s, *B* = 141) or spatially variable hematocrit and nonlinear Pries-Secomb blood rheology [hematocrit II, where the effective viscosity η(*H*) and solute carrying capacity *B*(*H*) vary across the network]. While the Fåhræus-Lindqvist effect can be expected to lower the net resistance of flow through the network, enhancing *N* for a given Δ*P*, the hematocrit reduction in smaller vessels due to plasma skimming reduces their oxygen carrying capacity. Figure 5C shows how, for specimen 1, the two effects are predicted to counteract, leading to modest net impact on overall oxygen transport, supporting the use of the Newtonian model and, furthermore, preserving the predictive power of the scaling relationship (*3*) in the discrete network model. However, the impact of the solute carrying capacity is significant (Fig. 5C): Setting *B* = 1 (hematocrit III) to eliminate the effect of solute binding to hemoglobin substantially reduces *N* compared with hematocrit I and hematocrit II, particularly under flow-limited conditions.

We also used the discrete network model to probe the sensitivity of oxygen transport to removal (or temporary blockage) of individual vessels. We calculated distributions of network oxygen transfer *N* when individual capillaries of specimen 1 are removed from the network (excluding those very close to the inlet). Removal of a single vessel reduces the overall network transfer by no more than 10% (see Fig. 5D), demonstrating the robustness of the network to the occlusion of individual capillaries.

## DISCUSSION

This study demonstrates how, despite highly variable network geometries, solute transfer between maternal and fetal circulations in a terminal villus can be characterized effectively using two dimensionless parameters (the diffusive capacity ratio μ and the Damköhler number Da; see Eqs. 1 and 2), which, in turn, depend on three geometry-dependent dimensional quantities (the total centerline length of capillaries within a network *L*_c_, the diffusive length scale L relating capillary and villus geometry, and the network flow resistance R). These can be extracted from microscopy images via standard tools (finite-element analysis and image skeletonization) and provide a computational generalization for disordered tissues of the classical Krogh cylinder approach. These variables reveal scaling relationships that hold both at the network and capillary levels: The appropriate choices of μ and Da lead to a near collapse of transport behavior across multiple terminal villi (Fig. 2B), as well as for individual capillaries within a villus network (Fig. 4B). Furthermore, the algebraic approximation Eq. 3 compactly summarizes the transport capacity of a villus. Its transparent dependence on physical parameters gives immediate insights into the physical and geometric determinants of solute transport, and its economy makes it attractive as a component in future multiscale models of placental function.

The model readily describes transfer of a variety of passively transported solutes. Varying diffusion coefficients and the binding capacity to hemoglobin influences μ and Da, revealing solutes that are predominantly flow or diffusion limited (table S1). The wide spread of parameter values illustrated in Fig. 3 (Da spans four orders of magnitude) emphasizes how flow- and diffusion-limited transport are likely to occur concurrently in a single villus for different solutes (*29*). It remains to be seen whether the relatively modest variation in μ compared to Da (Fig. 3) for oxygen and other mobile solutes indicates a possible robust design feature of fetoplacental microvasculature, which could be mediated in the developing placenta by the dynamic balance of angiogenesis and vascular pruning (*32*).

A 1D discrete network model (Fig. 5) offers a level of detail intermediate between the full 3D computational and algebraic regression (Eq. 3) approximations, enabling the analysis of fetoplacental transport performance at minimal computational and image processing costs. The discrete network model matches the predictions of the computational model in the physiological range of capillary pressure drops (Fig. 5, A and B); however, it overestimates the uptake flux for fast flows (in the diffusion-limited transport regime) because of its neglect of diffusive shielding, i.e., spatial interaction between neighboring capillaries (e.g., see Fig. 4D). The diffusive shielding is captured in 3D via L by integrating over the whole tissue domain, extending prior studies in 2D (*20*). Likewise, the discrete model overestimates the network flow resistance and, thus, underestimates the uptake flux at small pressure drops (in the flow-limited transport regime) due to the strong (fourth-power) sensitivity of resistance on capillary radii, which are more accurately captured by the integral resistance R of the 3D computational model.

The present model exploits emerging anatomical data for terminal villi but has some significant limitations. Our calculations over a discrete vessel network using the Pries-Secomb model (*31*), which characterizes hematocrit distributions in individual cylindrical vessels, suggest that the effect of non-Newtonian blood rheology on oxygen transport is modest (Fig. 5C) and that the network itself is robust to occlusions of individual vessels (Fig. 5D), which may occur transiently due (for example) to red blood cells lingering at network bifurcations (*33*). These predictions await confirmation through more detailed theoretical studies that describe blood rheology in complex geometrical domains, and suitable experimental observations. We have not accounted for uptake of solutes by the placental tissue itself, which will be a significant feature for solutes such as oxygen (and which could shift the transport into a more flow-limited regime); the predicted fluxes must therefore be treated as upper bounds until future studies address this feature in more detail. We have also encountered a common problem in simulating flows through microvascular networks, namely, in reliably identifying inlet and outlet vessels. This choice influences vessels that may serve as donors or recipients when countercurrent effects arise in the flow-limited regime (Fig. 4); however, the choice has negligible impact on net transport in the diffusion-limited regime. We have also oversimplified the supply of solute at the villus surface; this will be influenced by local features of the flow of maternal blood in the intervillous space. The model also assumes negligible interstitial flow in the villous tissue and does not account for transport of certain solutes via paracellular channels or energy-dependent membrane transporters (*3*, *23*). Last, our model does not explicitly account for nonlinear oxygen-hemoglobin binding kinetics [the effects of which are evaluated in (*26*)] and the particulate nature of capillary blood flow that could result in subtle spatial oxygen gradients [e.g., see (*34*) for an extensive overview]. While our modeling framework provides a robust qualitative description of transport in complex microvascular networks for a wide variety of solutes, it requires further quantitative refinement in future studies.

A key message of this study is that, despite the significant variability in the shapes of individual capillaries within a terminal villus, the overall capacity of the villus to transport passive solutes can be captured using three integrated quantities (*L*_c_, L, and R), which, to some extent, average out intrinsic variations. It remains to be seen to what extent local features such as isolated “hotspots” of transfer (where well-perfused capillaries lie very close to the villus surface, for example) might correlate with features of the external maternal flow, or the distribution of transporters in the villus membrane. These features may lead to nontrivial coupling between fetal and maternal flow distributions (*3*). Once suitable imaging data become available, it will be of particular interest to explore both intra- and interplacental variability and to examine how pathologies that disrupt the structure of terminal villi affect their function.

In summary, our analysis demonstrates how a judicious choice of dimensionless variables, incorporating relevant integral determinants of geometric microstructure, reveals robust relationships characterizing physiological function. We anticipate that the framework we propose for assessing fetoplacental solute transport performance can usefully be extended to other complex microvascular systems.

## MATERIALS AND METHODS

The specimens were taken from two different peripherial lobules of a normal human placenta delivered by cesarean section at term, as reported previously (*14*). The lobules were fixed at different fetoplacental fixation pressures [specimens 1 and 2 at 100 mmHg, specimens 3 and 4 at 30 mmHg; see (*14*)], and the samples within each lobule were randomly sampled. Full details of the image analysis, 3D flow and transport simulations, discrete network model, and sensitivity analysis are provided in the Supplementary Materials.

## Supplementary Material

http://advances.sciencemag.org/cgi/content/full/5/4/eaav6326/DC1

Download PDF

## SUPPLEMENTARY MATERIALS

Supplementary material for this article is available at http://advances.sciencemag.org/cgi/content/full/5/4/eaav6326/DC1 Section S1. Image analysis and network statistics Section S2. Computational model Section S3. Transport in a single cylindrical capillary Section S4. A discrete model for transport in a capillary network Fig. S1. Geometric statistics for terminal villus specimens. Fig. S2. Surfaces on which boundary conditions are imposed. Fig. S3. Shear stress distribution in a capillary network. Fig. S4. A schematic of a capillary network segment. Table S1. Characteristic parameters for various passively transported solutes. References (*35*–*47*)
