# Supplementary material for: Physical and geometric determinants of transport in fetoplacental microvascular networks
Source: Sci Adv. 2019 Apr 17;5(4):eaav6326. doi: 10.1126/sciadv.aav6326 (PMC6469945; doi:10.1126/sciadv.aav6326)
Supplement: http://advances.sciencemag.org/cgi/content/full/5/4/eaav6326/DC1 [file supp_5_4_eaav6326__index.html]

Science Advances | Science Advances

## Supplementary Materials

**This PDF file includes:**

- Section S1. Image analysis and network statistics
- Section S2. Computational model
- Section S3. Transport in a single cylindrical capillary
- Section S4. A discrete model for transport in a capillary network
- Fig. S1. Geometric statistics for terminal villus specimens.
- Fig. S2. Surfaces on which boundary conditions are imposed.
- Fig. S3. Shear stress distribution in a capillary network.
- Fig. S4. A schematic of a capillary network segment.
- Table S1. Characteristic parameters for various passively transported solutes.
- References (*35*–*47*)

Download PDF

**Files in this Data Supplement:**

- Adobe PDF - aav6326\_SM.pdf
