# Supplementary material for: Physical and geometric determinants of transport in fetoplacental microvascular networks
Source: Sci Adv. 2019 Apr 17;5(4):eaav6326. doi: 10.1126/sciadv.aav6326 (PMC6469945; doi:10.1126/sciadv.aav6326)
Supplement: Download PDF [file aav6326_SM.pdf]

Science Advances
AAAS

advances.sciencemag.org/cgi/content/full/5/4/eaav6326/DC1

Supplementary Materials for

Physical and geometric determinants of transport in fetoplacental microvascular networks

Alexander Erlich, Philip Pearce, Romina Plitman Mayo, Oliver E. Jensen, Igor L. Chernyavsky*

*Corresponding author. Email: igor.chernyavsky@manchester.ac.uk

Published 17 April 2019, *Sci. Adv.* **5**, eaav6326 (2019)
DOI: 10.1126/sciadv.aav6326

This PDF file includes:

Section S1. Image analysis and network statistics
Section S2. Computational model
Section S3. Transport in a single cylindrical capillary
Section S4. A discrete model for transport in a capillary network
Fig. S1. Geometric statistics for terminal villus specimens.
Fig. S2. Surfaces on which boundary conditions are imposed.
Fig. S3. Shear stress distribution in a capillary network.
Fig. S4. A schematic of a capillary network segment.
Table S1. Characteristic parameters for various passively transported solutes.
References (*35–47*)

This Supplement provides further details on the image analysis (Sec. S1), the governing equations and methodology used in 3D computations (Sec. S2), the asymptotic model of transport in a single vessel that motivates the regression equation (Sec. S3), the discrete network transport model (Sec. S4) and its use in assessing the impact of nonlinear blood rheology and network heterogeneity. The associated structural datasets and computational codes are publicly available in the Figshare repository: `https://doi.org/10.6084/m9.figshare.7016303`.

Section S1. Image analysis and network statistics

The images used here comprise four sets of smooth 3D meshes of fetal vasculature and the accompanying villous membrane (fig. S1A), segmented from stained confocal microscopy data (Fig. 1B) as described previously [14]. Image dimensions are approximately $(250 \times 250 \times 150)\,\mu$m.

The watershedding algorithm *AutoSkeleton* of FEI Amira™ 6.4 was used to skeletonize capillary centerlines from 3D meshes, as illustrated in Fig. 1D. Having identified branching points, each network can be represented as a graph (for example, a 2D projection of the 37-segment graph for Specimen 1 is illustrated in the inset to Fig. 5D).

We extracted geometrical statistics for each capillary branch (capillary length, and vessel-averaged minimal distances from centerline to capillary surface and from centerline to villous surface) using Wolfram Mathematica® 11.2. As fig. S1B illustrates, the vessel-averaged minimal distances across all specimens from centerline to capillary surface is $8.0\,\mu$m, and from centerline to villous surface it is $17.9\,\mu$m.

Section S2. Computational model

Governing equations

In simulations, we model fetal blood flow using the Stokes equations

$$\eta \nabla^2 \boldsymbol{u} = \nabla p, \qquad \nabla \cdot \boldsymbol{u} = 0 \tag{S1}$$

Here $\boldsymbol{u}$ is the fluid velocity field, p the fluid pressure and η the dynamic viscosity of fetal blood, which is treated as Newtonian in 3D simulations; we take $\eta = 2 \times 10^{-3}$ Pa·s (appropriate for blood with 48% hematocrit in a $20\,\mu$m vessel; see [26]). We address the effects of nonlinear blood rheology in Sec. S4 below.

The solute concentration c in blood is assumed to obey the linear advection-diffusion equation

$$B\mathbf{u} \cdot \nabla c = D_{\mathrm{p}} \nabla^2 c \tag{S2}$$

where D_{p} is the solute diffusion coefficient in plasma. The parameter $B = 1$ for most solutes, but for species that bind to hemoglobin B quantifies the facilitated transport by red blood cells [21, 26]. For example, for oxygen [21, 26]

$$B = 1 + c_{\max} K k_{\mathrm{hn}} / \rho_{\mathrm{bl}} \approx 141 \tag{S3}$$

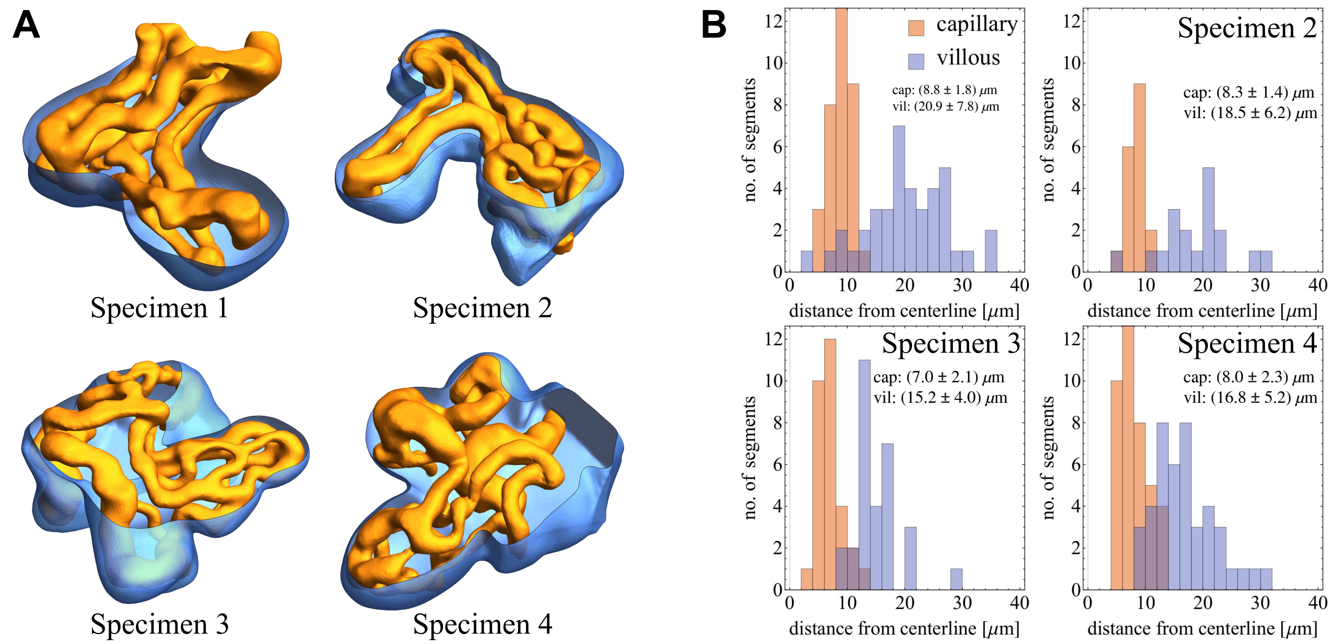

Fig. S1. **Geometric statistics for terminal villus specimens. A** Four segmented terminal villi, showing capillary surface (rendered in yellow) and syncytiotrophoblast (blue). **B** Vessel-averaged minimal distance between centerline and Γ_{cap}, as well as between centerline Γ_{vil}. The vessel-averaging consists of discretizing each vessel centerline into 50-100 points, calculating the minimal distance to the respective surface (Γ_{cap} or Γ_{vil}), and taking the mean value of said minimal distances, collapsing every vessel to a single distance value. The distributions of vessel-averaged minimal distances from centerline to capillary surface (red) and to villous surface (blue) are shown for Specimens 1–4; each network comprises between 18 and 43 vessels. The mean and standard deviation for capillary and villous distances of each specimen are given in the figures in the form (mean ± SD) μm. Across all specimens, the vessel-averaged minimal distances from centerline to capillary surface is 8.0 μm, and from centerline to villous surface it is 17.9 μm.

where c_{max} is the oxygen content of fetal blood at full saturation, K is the gradient of the linearized fetal oxygen-hemoglobin dissociation curve [26], k_{hn} is the Henry's law coefficient and ρ_{bl} is the density of blood. In villous tissue, solute transport is governed by the diffusion equation

$$D_{\mathrm{t}}\nabla^2 c = 0 \tag{S4}$$

where D_t is the solute diffusion coefficient in tissue. Linearity of Eqs (S2), (S4) is convenient in allowing solute fields to be rescaled to describe transport of solutes with different concentrations.

Boundary conditions

The surfaces bounding the domains in which Eqs (S1), (S2), (S4) are solved are illustrated in fig. S2A,B. For the Stokes problem Eq. (S1), blood enters through the inlet surface Γ_{in} and leaves via Γ_{out}, driven by a pressure difference ΔP imposed between inlet and outlet. A no-slip condition is imposed on the capillary surface Γ_{cap}. The boundary conditions on the flow are therefore

$$p = \Delta P \quad \text{on} \quad \Gamma_{\mathrm{in}} \tag{S5}$$
$$p = 0 \quad \text{on} \quad \Gamma_{\mathrm{out}} \tag{S6}$$
$$\boldsymbol{u} = 0 \quad \text{on} \quad \Gamma_{\mathrm{cap}} \tag{S7}$$

Fetal blood is assumed to enter solute-free at the inlet Γ_{in} and zero diffusive solute flux is imposed at the outlet Γ_{out}. Although it is difficult to reliably identify inlet and outlet vessels from the reconstructed geometry alone, the choice

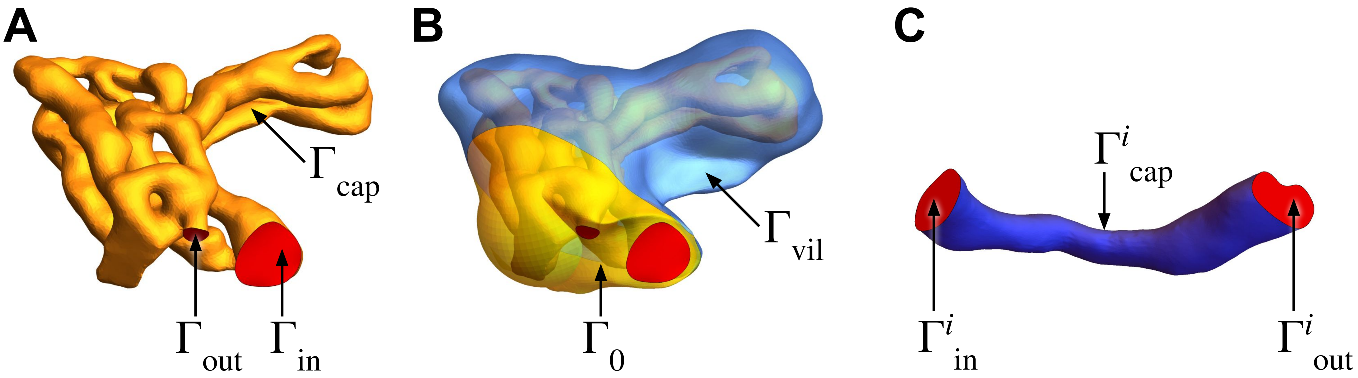

Fig. S2. Surfaces on which boundary conditions are imposed. Relevant surfaces for the Stokes problem (**A**, see Eqs (S5)–(S7)) and additional surfaces for the advection-diffusion solute transport problem and computation of carrying capacity $N_{\max}$ (**B**, see Eqs (S8)–(S14)) are shown. When integrating solute transport fluxes over individual capillaries, the labelling convention shown in **C** is used.

has no impact on flow resistance when there is a single inlet and outlet, nor on the maximum diffusive flux (see below) in the diffusion-limited regime. The solute concentration and diffusive solute flux are assumed continuous across the internal boundary Γ_{cap}. The maternal solute concentration $c = c_{\mathrm{mat}}$ is imposed on the villous surface Γ_{vil} and no diffusive flux is imposed between the inlet/outlet and the villous surface (on Γ_0) to avoid artificial sharp gradients. Together, the external boundary conditions on the solute are

$$c = 0 \quad \text{on} \quad \Gamma_{\mathrm{in}} \tag{S8}$$
$$\boldsymbol{n} \cdot \nabla c = 0 \quad \text{on} \quad \Gamma_{\mathrm{out}}, \Gamma_0 \tag{S9}$$
$$c = c_{\mathrm{mat}} \quad \text{on} \quad \Gamma_{\mathrm{vil}} \tag{S10}$$

For oxygen, we assume $c_{\mathrm{mat}} \approx 0.07\,\mathrm{mol/m^3}$ [26].

Net solute transfer

The net solute transfer rate N of the network is defined as the diffusive flux across Γ_{vil} or equivalently across Γ_{cap}. As diffusive fluxes across Γ_{in} are very small for the parameters of interest, N is well approximated as the advective flux leaving the flow domain capillary network

$$N = \iint_{\Gamma_{\mathrm{out}}} B\, c\, \boldsymbol{n} \cdot \boldsymbol{u} \,\mathrm{d}A \tag{S11}$$

where $\boldsymbol{n}$ is the unit outward normal to Γ_{out}. We test mass conservation by comparing the advective flux Eq. (S11) over the capillary domain (Γ_{out}) with the diffusive flux over the villous domain (Γ_{in} and Γ_{vil}) to validate the numerical implementation.

The maximum diffusive flux

The maximum diffusive flux (or carrying capacity) $N_{\max}$ corresponds to the net solute flux arising when the flow is sufficiently strong for the inlet condition $c = 0$ to apply across Γ_{cap}. It can be calculated by solving Eq. (S4) over the villous tissue domain with boundary conditions

$$c = 0 \quad \text{on} \quad \Gamma_{\mathrm{cap}} \tag{S12}$$
$$\boldsymbol{n} \cdot \nabla c = 0 \quad \text{on} \quad \Gamma_0 \tag{S13}$$
$$c = c_{\mathrm{mat}} \quad \text{on} \quad \Gamma_{\mathrm{vil}} \tag{S14}$$

and evaluating the diffusive flux across the capillary surface

$$N_{\max} = -\iint_{\Gamma_{\mathrm{cap}}} D_{\mathrm{t}}\, \boldsymbol{n} \cdot \nabla c \,\mathrm{d}A \tag{S15}$$

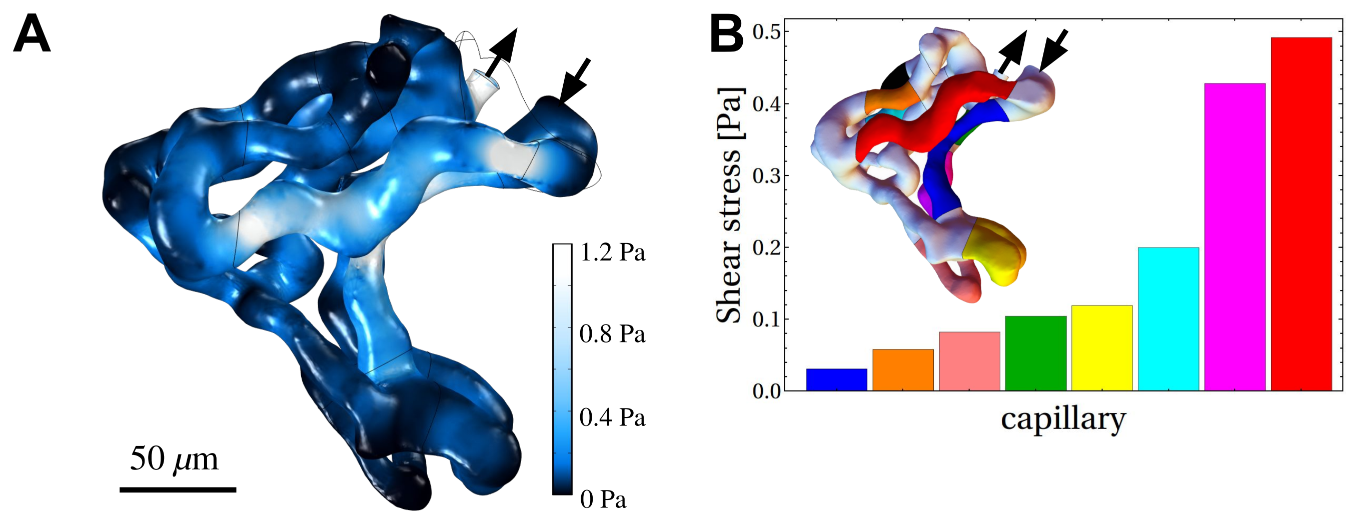

Fig. S3. **Shear stress distribution in a capillary. A** Predicted shear stresses in Specimen 1 shown for an inlet-outlet pressure drop of $\Delta P = 40\,\mathrm{Pa}$. The highest wall shear stresses (white, around 1.2 Pa) occur where capillaries are thin and flow speeds are greatest, e.g. at the outlet (near the outward-pointing arrow). **B** Average shear stresses of the nine capillaries (inset) discussed in Fig. 4A–C in the main text.

The parameter characterizing integrated exchange area over exchange distance is then defined by

$$\mathcal{L} = N_{\mathrm{max}} \,/\, (D_{\mathrm{t}}\, c_{\mathrm{mat}}) \tag{S16}$$

Numerical implementation

We used COMSOL Multiphysics® 5.3a to solve the coupled flow and transport problems defined above. For the Stokes problem in Figs 4, 5A,B and S3, we used the *Creeping Flow* module, calculating the solution on capillary meshes of approximately 5.6 million tetrahedral elements. To calculate the concentration field in these figures, we used the *Transport of Diluted Species* module on meshes of approximately 61.6 million tetrahedral elements. To ensure that concentration boundary layers (should they arise) and fine details of the mesh (such as local near-contact of villous and capillary meshes) were resolved, we performed a mesh convergence analysis. For the most intricate mesh (Specimen 3), an almost nine-fold increase from approximately 7.6 to 65.6 million tetrahedral elements changed the net uptake flux N at $\Delta P = 40\,\mathrm{Pa}$ by less than 3%.

To calculate fluxes, we used *Accurate Fluxes* in COMSOL (tds.ncflux_c and tds.ndflux_c for advective and diffusive fluxes, respectively) and ensured that advective fluxes integrated over closed domains match. In doing so, we evaluated the net flux over an individual capillary i (see fig. S2C) using

$$N_i = \iint_{\Gamma^i_{\mathrm{out}}} B\, c\, \boldsymbol{n} \cdot \boldsymbol{u}\, \mathrm{d}A - \iint_{\Gamma^i_{\mathrm{in}}} B\, c\, \boldsymbol{n} \cdot \boldsymbol{u}\, \mathrm{d}A \tag{S17}$$

and evaluated N^i_{max} for an individual capillary via the diffusive flux over its capillary wall Γ^i_{cap}. In the case of net flux computations over the entire network (Fig. 2 in the main text), we calculate N by integrating over the entire villous surface to minimise error introduced by very small outlet surfaces.

The data underlying Figs 2 and 3 of the main text were produced with comparable mesh quality for all four specimens. Stokes flow on the capillary domain was solved on meshes with between 0.3 to 1.2 million tetrahedral elements. Transport was solved on the villous and capillary domains on meshes with between 4.1 and 22.4 million tetrahedral elements. For Specimen 1, the comparison between the net solute transfer across the network calculated at high resolution (61.6 million tetrahedral element mesh for transport problem, as used in Fig. 4) and low resolution (15.0 million tetrahedral element mesh for transport problem, as used in Fig. 2) led to a maximal relative error of 5.2% at a very high pressure drop ($\Delta P = 2725.2\,\mathrm{Pa}$); at a physiological pressure drop of $\Delta P = 40\,\mathrm{Pa}$, the relative error was 0.6%.

Table S1. Characteristic parameters for various passively transported solutes. The constant B describes the solute carrying capacity by the red blood cells. The solute diffusivities in blood plasma and in villous tissue (where the solute is dissolved in water) are D_p and D_t respectively. The Damköhler and diffusive capacity numbers relative to oxygen values are $\mathrm{Da}_\mathrm{rel} \equiv D_\mathrm{t}^\mathrm{solute} B^\mathrm{oxygen} / (D_\mathrm{t}^\mathrm{oxygen} B^\mathrm{solute})$ and $\mu_\mathrm{rel} \equiv D_\mathrm{t}^\mathrm{solute} / D_\mathrm{p}^\mathrm{solute}$ respectively. Data are taken (with most D_p values given in literature at 25°C) or estimated from [a][29, 36], [b][37], [c][38]. [d][39], [e][40, 41], [f][42–44],[g][26], [h][45].

Solute	B	D_p $\times 10^{-9}$ [m²/s]	D_t $\times 10^{-9}$ [m²/s]	$(\mathrm{Da}_\mathrm{rel})^{-1}$	μ_rel
carbon monoxide (CO)	$\sim 10^4$ [a]	2 [b]		$\sim 10^2$	1
mannitol	1	0.7 [c]	$\sim (10^{-4} - 10^{-3})$ [d]	$\sim 10 - 10^2$	$\sim 10^{-3} - 10^{-2}$
fructose	1	0.7 [e]	$\sim (10^{-4} - 10^{-3})$ [f]	$\sim 10 - 10^2$	$\sim 10^{-3} - 10^{-2}$
glucose	1	0.7 [e]	$\sim (10^{-3} - 10^{-2})$ [f]	$\sim 1 - 10$	$\sim 10^{-2} - 10^{-1}$
oxygen (O_2)	≈ 140 [g]	2 [b]		1	1
carbon dioxide (CO_2)	$\sim (1 - 10)$ [h]	1.9 [b]		$\sim 10^{-2} - 10^{-1}$	1
nitrous oxide (N_2O)	1	2.6 [c]		$\sim 10^{-2}$	1
urea	1	1.4 [b]		$\sim 10^{-2}$	1
ethanol	1	1.2 [e]		$\sim 10^{-2}$	1
caffeine	1	0.8 [c]		$\sim 10^{-2}$	1

Inlets, outlets and boundary surfaces

The three-dimensional mesh data of capillary and villous surfaces has a number of imperfections and imaging artefacts that add a subjective component to the identification of boundary conditions. The Specimen 1-4 meshes have between three and five candidate locations for inlets and outlets, and we made our choice of inlet and outlet on a case-by-case basis: in Specimen 1, 3 and 4 we identified one likely inlet and one likely outlet per specimen, and made slight modifications by locally adding small hemispheres to the villous surface at the discarded inlet/outlet candidate locations. These modifications ensure that apart from at the inlet and outlet, the villous surface does not come unnecessarily close to the capillary surface. Another imperfection of the imaging data arose due to the depth limitations of confocal microscopy, which sometimes makes it unclear if a part of the villous surface was originally in contact with maternal blood or resulted from an artificially cut-off internal boundary on which unphysiological oxygen exchange could occur. In the latter case, a no-flux boundary condition is applied as appropriate (Γ_0 in fig. S2). We identified the no-flux planes vs. exchange planes according to our best judgement. Comparisons between different choices of no-flux planes revealed differences in $\mathcal{L}$ of up to 12%.

Shear stress distributions

In addition to the results reported in Figs 2–4 of the main text, the computational model provides detailed maps of predicted shear stress within capillaries (fig. S3A). For a network pressure drop ΔP of 40 Pa, the shear stress is everywhere below a maximum of approximately 1.2 Pa; for comparison, Olesen et al. [35] estimated a physiological shear stress range between 0.5 Pa to 2 Pa in arterioles of comparable diameter to those encountered here. The shear stress at any location within the network is linearly proportional to ΔP under a Newtonian Stokes flow approximation, suggesting that an increase of ΔP to around 100 Pa remains within a physiological range. Regions of locally elevated shear stress are found at constrictions and in vessels carrying greater flow, for example near the inlet or outlet. The variation in average shear stress between vessels (fig. S3B) was notable, indicating local variations in flow resistance. However, these results depend on the specimen fixation pressure and the choice of flow rheology model (see Sec. S4 below and Figs 4 and 5 in the main text.).

Model parameters for passively transported solutes

The developed framework readily extends to a variety of relatively small and mobile solutes. Table S1 summarises and estimates key transport parameters, specifically effective advection-enhancement factors B, plasma D_p and tissue D_t diffusivities, as well Damköhler and diffusive capacity numbers (Da_rel and μ_rel) relative to oxygen values.

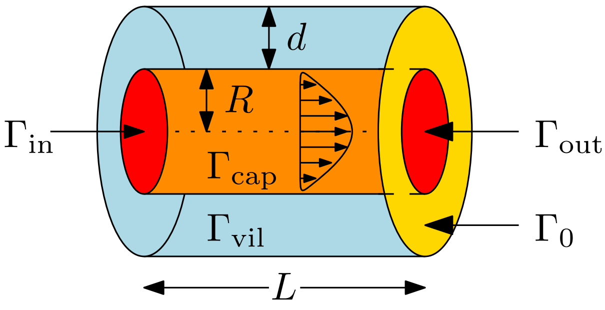

Fig. S4. A schematic of a capillary network segment. A capillary is represented in the discrete network model by a cylinder with Poiseuille flow and a surrounding cylindrical shell representing the villous domain. The boundary surfaces are labeled in the convention of fig. S2.

Section S3. Transport in a single cylindrical capillary

We now motivate the form of the regression equation, Eq. (3) in the main text, by analysing transport in a single capillary. We assume axisymmetry, denoting parameters in this special case with a circle superscript.

Consider a cylindrical feto-placental capillary of length L and radius R within an annular villous volume of thickness d (fig. S4). In cylindrical coordinates, the flow problem Eq. (S1), (S5)–(S7) has the familiar Poiseuille solution for the axial velocity

$$u\left(r\right) = u_{\text{max}}\left(1 - \frac{r^2}{R^2}\right), \quad u_{\text{max}} = \frac{\Delta P R^2}{4\eta L} \tag{S18}$$

The cross-sectionally averaged velocity is $\langle u \rangle = \frac{1}{A}\iint u \, \mathrm{d}A = u_{\text{max}}/2$, where A is the cross-section area. The volume flux $q = \iint u \, \mathrm{d}A$ is related to the pressure drop ΔP across the capillary via $\Delta P = \mathcal{R}^{\circ} q$ with the Poiseuille resistance

$$\mathcal{R}^{\circ} = \frac{8\eta L}{\pi R^4} \tag{S19}$$

The advection-diffusion problem given by Eqs (S2), (S8)–(S10) becomes

$$Bu\frac{\partial c}{\partial z} = D_{\text{p}}\left[\frac{1}{r}\frac{\partial}{\partial r}\left(r\frac{\partial c}{\partial r}\right) + \frac{\partial^2 c}{\partial z^2}\right] \tag{S20}$$

$$0 = D_{\text{t}}\left[\frac{1}{r}\frac{\partial}{\partial r}\left(r\frac{\partial c_{\text{t}}}{\partial r}\right) + \frac{\partial^2 c_{\text{t}}}{\partial z^2}\right] \tag{S21}$$

Here we use c_{t} to denote the solute concentration in villous tissue. The boundary conditions are

$$c = 0 \qquad \text{at } z = 0 \tag{S22}$$

$$\frac{\partial c}{\partial z} = 0 \qquad \text{at } z = L \tag{S23}$$

$$c_{\text{t}} = c_{\text{mat}} \qquad \text{at } r = R + d \tag{S24}$$

$$D_{\text{p}}\frac{\partial c}{\partial r} = D_{\text{t}}\frac{\partial c_{\text{t}}}{\partial r} \qquad \text{at } r = R \tag{S25}$$

$$c = c_{\text{t}} \qquad \text{at } r = R \tag{S26}$$

$$\frac{\partial c}{\partial r} = 0 \qquad \text{at } r = 0 \tag{S27}$$

Neglecting axial diffusion in Eq. (S21), we can obtain an explicit solution for c_{t} in terms of c, allowing us to write the full problem in terms of the capillary concentration c only. The Neumann condition Eq. (S25) becomes a Robin condition

$$\frac{\partial c}{\partial r} = \mu_{\circ}\left(\frac{c_{\text{mat}} - c}{R}\right) \quad \text{at } r = R \tag{S28}$$

with the diffusive capacity

$$\mu_{\circ} = \frac{D_{\text{t}}/D_{\text{p}}}{\log(1 + d/R)} \tag{S29}$$

Setting $c = 0$ in Eq. (S28) and integrating the diffusive flux over Γ_{cap}, it follows that $N^{\circ}_{\text{max}} = 2\pi D_{\text{t}}\, c_{\text{mat}}\, L/\log(1{+}d/R)$.

Asymptotic approximation

Introducing the non-dimensional variables

$$\Pi = \frac{B\,R\,u_{\text{max}}}{\sqrt{\mu_\circ}D_{\text{p}}}, \quad \alpha = \frac{L}{R}, \quad \hat{r} = \frac{r}{R}, \quad \hat{z} = \frac{z}{R}, \quad \hat{c} = \frac{c}{c_{\text{mat}}} \tag{S30}$$

the problem is specified in terms of $\mu_\circ$, a modified Péclet number Π and the tube aspect ratio α as

$$\Pi\sqrt{\mu_0}\left(1-\hat{r}^2\right)\frac{\partial \hat{c}}{\partial \hat{z}} = \frac{1}{\hat{r}}\frac{\partial}{\partial \hat{r}}\left(\hat{r}\frac{\partial \hat{c}}{\partial \hat{r}}\right) + \frac{\partial^2 \hat{c}}{\partial \hat{z}^2} \tag{S31}$$

with boundary conditions

$$\hat{c} = 0 \qquad \text{at } \hat{z} = 0 \tag{S32}$$

$$\frac{\partial \hat{c}}{\partial \hat{r}} = 0 \qquad \text{at } \hat{r} = 0 \tag{S33}$$

$$\frac{\partial \hat{c}}{\partial \hat{z}} = 0 \qquad \text{at } \hat{z} = \alpha \tag{S34}$$

$$\frac{\partial \hat{c}}{\partial \hat{r}} = \mu_\circ(1-\hat{c}) \quad \text{at } \hat{r} = 1 \tag{S35}$$

We now demonstrate how diffusion-limited and strongly or weakly flow-limited regimes can be obtained from this boundary-value problem.

When the diffusive capacity is low ($\mu_\circ \ll 1$), radial diffusion over a long domain suppresses transverse concentration gradients. Following [46], we scale the axial coordinate by $\sqrt{\mu_\circ}$ and approximate the concentration profile as well-mixed, using

$$\hat{z} = \frac{\overline{z}}{\sqrt{\mu_\circ}}, \qquad \hat{c} = \hat{c}_0\left(\overline{z}\right) + \mu_\circ \hat{c}_1\left(\overline{z}, \hat{r}\right) + \mathcal{O}\left(\mu_\circ^2\right) \tag{S36}$$

The non-dimensional problem Eq. (S31) then becomes

$$\Pi\left(1-\hat{r}^2\right)\frac{\partial \hat{c}_0}{\partial \overline{z}} = \frac{1}{\hat{r}}\frac{\partial}{\partial \hat{r}}\left(\hat{r}\frac{\partial \hat{c}_1}{\partial \hat{r}}\right) + \frac{\partial^2 \hat{c}_0}{\partial \overline{z}^2} \tag{S37}$$

Integrating Eq. (S37) over the cross-section and imposing boundary conditions we obtain the ordinary differential equation

$$\frac{\Pi}{4}\hat{c}_0'\left(\overline{z}\right) = \tfrac{1}{2}\hat{c}_0''\left(\overline{z}\right) + 1 - \hat{c}_0\left(\overline{z}\right), \quad \hat{c}_0\left(0\right) = 0, \quad c_0'\left(\overline{\alpha}\right) = 0 \tag{S38}$$

where $\overline{\alpha} = \sqrt{\mu_\circ}\alpha$. The solution $\hat{c}_0$ to this boundary value problem can be integrated as $N \propto \mu_\circ\left(\int_{\hat{z}=0}^{\alpha} \hat{c}_0 \mathrm{d}\hat{z} - \alpha\right)$ to find the net uptake. When axial diffusion is weak ($\Pi \gg 1$), we find

$$N \approx N_{\text{FL-DL}} \equiv N^{\circ}_{\text{max}}\,\text{Da}_\circ^{-1}\left(1 - e^{-\text{Da}_\circ}\right) \tag{S39}$$

where the relevant inverse Damkhler number is

$$\text{Da}_\circ^{-1} = \frac{D_{\text{t}}}{D_{\text{p}}}\frac{\Pi}{4\sqrt{\mu_\circ}\alpha} \tag{S40}$$

Eq. (S39) encompasses the strongly flow-limited regime $N \approx N^{\circ}_{\text{max}}\text{Da}_\circ^{-1}$ when $\text{Da}_\circ \gg 1$ and the diffusion-limited regime $N \approx N^{\circ}_{\text{max}}$ when $\text{Da}_\circ \ll 1$.

In the strongly flow-limited regime, the assumption of a nearly flat concentration profile Eq. (S36) is no longer viable, as concentration boundary layers form in a corner region near the inlet of the tube. Instead the Lévêque approximation

must be employed, which requires a transformation into the boundary layer coordinate system [37, 46]. This allows us to recover the weakly flow-limited regime

$$N_{\mathrm{WFL}} = N^{\circ}_{\mathrm{max}} \alpha_{\mathrm{c}} \mathrm{Da}_{\circ}^{-1/3} \mu_{\circ}^{-2/3} \tag{S41}$$

where $\alpha_{\mathrm{c}} \approx 5.5$. An approximation for N across all physical regimes can then be obtained from a harmonic mean of $N_{\text{FL-DL}}$ and N_{WFL}

$$N^{-1} = N^{-1}_{\text{FL-DL}} + N^{-1}_{\mathrm{WFL}} \tag{S42}$$

This predicts N in terms of the geometric parameters R, d and L, the material parameters η, B, D_{p} and D_{t}, the imposed pressure drop ΔP and the concentration difference c_{mat}. The empirical regression equation Eq. (3) in the main text, generalizes this approach to the whole network.

Section S4. A discrete model for transport in a capillary network

In order to explore the effect of hematocrit on solute transport in feto-placental capillary networks, and to test the system for sensitivity to occlusion of single vessels, we develop a discrete network model that resolves individual capillaries as elements of a graph. We approximate solute transport by adapting the modified Krogh cylinder formulation in Sec. S3 above, ensuring conservation of fluid and solute at all nodes in the capillary network. We test the reduction from a continuous formulation using partial differential equations (Sec. S2) to a discrete (algebraic) representation before using the simplified model to evaluate the distribution of hematocrit in the network, calculated using the empirical law for plasma skimming from [31]. The distribution of hematocrit is used to calculate the effective viscosity in each vessel due to the Fåhræus–Lindqvist effect. We also test the sensitivity of the network to blockage of individual vessels.

The capillary network as a directed graph

Our low-order model for transport in a capillary network adapts and expands Strang's treatment of electrical circuits [47]. Consider a network having m segments (capillaries), each with an assigned orientation, and n nodes. To describe the relationship between nodal and segmental quantities, we introduce the $m \times n$ incidence matrix A. Its entries A_{ij} are either $+1$, 0 or -1, where 0 means that an edge and a node are not incident, $+1$ means that a directed edge points towards the node, -1 means that the edge points away from the node. It is helpful to introduce the downstream incidence matrix A_+ (in which all negative entries of A have been set to zero) and the upstream incidence matrix A_- (in which all positive entries of A have been set to zero) such that $\mathsf{A} = \mathsf{A}_- + \mathsf{A}_+$. Over all the segments we define a vector of scalar fluxes $\boldsymbol{q} = (q_1, \ldots, q_m)^T$, where $q_i > 0$ indicates that the flow direction in segment i matches the orientation of the segment i. Over the nodes we define vectors of scalar pressures $\boldsymbol{p} = (p_1, \ldots, p_n)^T$ and inlet concentrations $\boldsymbol{c} = (c_1, \ldots, c_n)^T$. $\mathsf{A}\boldsymbol{p}$ is then a vector of pressure differences, defined over directed segments.
Writing $\mathsf{B} = \mathrm{diag}\,(B_1, \ldots, B_m)$ as a diagonal matrix of advection boost coefficients and $\mathsf{Q} = \mathrm{diag}\,(q_1, \ldots, q_m)$ as a diagonal matrix of fluxes, we introduce the m-dimensional vector of advective fluxes over segments

$$\boldsymbol{n}^a = -\mathsf{B}\,\mathsf{Q}\,\mathsf{A}_- \boldsymbol{c} \tag{S43}$$

Defining transmural fluxes N_i for $i = 1, \ldots, m$ using the single tube results Eq. (S42) (in terms of three geometric parameters for each vessel and the pressure drop across it), we construct the diagonal matrix $\mathsf{N} = \mathrm{diag}\,(N_1, \ldots, N_m)$. Rescaling the fluxes to the relevant local concentrations, the m-dimensional vector of transmural diffusive fluxes is then

$$\boldsymbol{n}^d = \mathsf{N}\left(\mathbf{1} + c^{-1}_{\mathrm{mat}} \mathsf{A}_- \boldsymbol{c}\right) \tag{S44}$$

where $\mathbf{1} = (1, \ldots, 1)^T$ is an m-dimensional vector.

The discrete flow and transport problem over the network can then be written compactly as

$$\text{volume flux conservation} \qquad \mathsf{A}^T \boldsymbol{q} = \boldsymbol{f}_{\mathrm{ext}} \tag{S45}$$

$$\text{flow resistance} \qquad \mathsf{A}\boldsymbol{p} - \mathsf{R}\boldsymbol{q} = \mathbf{0} \tag{S46}$$

$$\text{advection-diffusion transport} \qquad \mathsf{A}^T \boldsymbol{n}^a + \mathsf{A}_+^T \boldsymbol{n}^d = \boldsymbol{g}_{\mathrm{ext}} \tag{S47}$$

Here $\boldsymbol{f}_{\text{ext}} = (-Q_{\text{ext}}, 0, \ldots, 0, Q_{\text{ext}})$ is an n-dimensional vector having first and last entries accounting for the scalar volume flux Q_{ext} entering and leaving the system. The system Eq. (S45) of n linear equations enforces conservation of volume flux at every node, which is coupled to the m-dimensional linear system Eq. (S46) describing Poiseuille's Law $\Delta P = \mathcal{R}^{\circ} q$ for the network; here $\mathsf{R} = \text{diag}(\mathcal{R}_1^{\circ}, \ldots, \mathcal{R}_m^{\circ})$. Finally, the m-dimensional linear system Eq. (S47) describes the transport, i.e. the balance of advective and diffusive fluxes. In total there are $n + 2m$ linear equations for the unknowns $\boldsymbol{p}$, $\boldsymbol{q}$ and $\boldsymbol{c}$. Boundary conditions can be imposed through the n-dimensional vectors $\boldsymbol{f}_{\text{ext}}$, $\boldsymbol{g}_{\text{ext}}$. A key assumption of the model is that concentration is fully mixed (i.e. has a radially independent profile) at every node.

In Fig. 5C of the main text, we compare the computational results of the discrete network model with the regression Eq. (3) applied to the whole network, which depends on the maximum achievable uptake flux $N_{\max}$ and flow resistance $\mathcal{R}$ of the discrete network. These quantities were computed directly from the discrete model (parameterized directly by the vessel-averaged statistics, fig. S1, rather than computational fluid dynamics results). We estimate $\mathcal{R}$ by calculating the flow rate at the inlet segment of the discrete network (which is equal to the flow rate at the outlet segment) and dividing by the applied inlet-outlet pressure drop. To compute $N_{\max}$, we apply a sufficiently high inlet-outlet pressure drop ΔP such that further increase in ΔP does not change the net uptake N by more than 0.01%, which is then used as the discrete network's $N_{\max}$.

Hematocrit and nonlinear rheology

Having established that the discrete network model provides a reliable representation of transport at the level of individual vessels (Fig. 5A,B), we now use it to explore the impact of nonlinear blood rheology on solute transport. This model accounts for plasma skimming, the Fåhræus effect and the Fåhræus–Lindqvist effect; to incorporate facilitated transport, we use a linearized oxygen-hemoglobin dissociation curve [21, 26].

The distribution of hematocrit in the network is calculated using the empirical law for plasma skimming from [31]. The fraction of hematocrit FQ_E entering a vessel at a bifurcation is found in terms of the fraction of blood flow FQ_B entering that vessel using

$$\text{logit } FQ_E = C_1 + C_2 \text{ logit}\left(\frac{FQ_B - X_0}{1 - 2X_0}\right) \tag{S48}$$

where logit $x \equiv \ln\left(x/\left(1-x\right)\right)$ and the parameter X_0 defines the minimal fractional blood flow required to draw red blood cells into the branch. The constants in Eq. (S48) are given by

$$C_1 = -6.96 \ln\left(\frac{R_1}{R_2}\right) / \left(2R_F\right), \quad C_2 = 1 + 6.98\left(\frac{1 - H_F}{2R_F}\right), \quad X_0 = \frac{0.4}{2R_F} \tag{S49}$$

where H_F and R_F are the hematocrit and the radius of the feeding vessel, R_1 is the radius of the vessel being considered and R_2 is the radius of the other vessel in the bifurcation (radii are measured in μm).

The distribution of hematocrit H is used to calculate the effective viscosity η in each vessel due to the Fåhræus–Lindqvist effect according to

$$\eta / \eta_{\text{p}} = 1 + \frac{e^{H\beta} - 1}{e^{0.45\beta} - 1}\left(110e^{-2.848R} + 3 - 3.45e^{-0.07R}\right) \tag{S50}$$

where $\beta = 4/\left(1 + \exp\left(-0.0593\left(2R - 6.74\right)\right)\right)$ and $\eta_{\text{p}} = 10^{-3}\,\text{Pa}\cdot\text{s}$ is the viscosity of plasma ($\eta \approx 2\eta_{\text{p}}$ in a vessel of radius $R = 10\,\mu$m for $H = 0.48$) [31]. The two steps above are implemented in the discrete model and iterated using a custom MathWorks MATLAB® R2016a code until the solution no longer changes, typically after less than 50 iterations. The MATLAB code was coupled with Wolfram Mathematica® 11.2 via the MATLink 1.1 package.
